# Impact of Early Conventional Treatment on Adult Bone and Joints in a Murine Model of X-Linked Hypophosphatemia

**DOI:** 10.3389/fcell.2020.591417

**Published:** 2021-02-18

**Authors:** Axelle Cauliez, Volha V. Zhukouskaya, Stéphane Hilliquin, Jérémy Sadoine, Lotfi Slimani, Corinne Miceli-Richard, Karine Briot, Agnès Linglart, Catherine Chaussain, Claire Bardet

**Affiliations:** ^1^Université de Paris, Laboratory Orofacial Pathologies, Imaging and Biotherapies URP 2496 and FHU-DDS-Net, Dental School, and Plateforme d’Imagerie du Vivant (PIV), Montrouge, France; ^2^INTEGRARE, Genethon, Inserm, Université d’Evry, Université Paris-Saclay, Evry, France; ^3^Centre de référence des maladies rares du métabolisme du calcium et du phosphate, Plateforme d’expertise maladies rares Paris Saclay, filière OSCAR, EndoRare and BOND ERN, Hôpital de Bicêtre, Le Kremlin-Bicêtre, France; ^4^Department of Rheumatology, Cochin Hospital, Université de Paris, Paris, France; ^5^Université Paris-Saclay, AP-HP, Service d’endocrinologie et diabète de l’enfant, Service de médecine des adolescents, Hôpital de Bicêtre, INSERM U1185, Le Kremlin-Bicêtre, France; ^6^AP-HP Reference Center for Rare Disorders of the Calcium and Phosphate Metabolism, Dental Medicine Department, Bretonneau Hospital, GHN, Paris, France

**Keywords:** rickets, osteomalacia, Hyp mice, phosphorus, conventional treatment, XLH, PHEX, fibroblast growth factor 23

## Abstract

X-linked hypophosphatemia (XLH) is the most common form of genetic rickets. Mainly diagnosed during childhood because of growth retardation and deformities of the lower limbs, the disease affects adults with early enthesopathies and joint structural damage that significantly alter patient quality of life. The conventional treatment, based on phosphorus supplementation and active vitamin D analogs, is commonly administered from early childhood to the end of growth; unfortunately, it does not allow complete recovery from skeletal damage. Despite adequate treatment during childhood, bone and joint complications occur in adults and become a dominant feature in the natural history of the disease. Our previous data showed that the *Hyp* mouse is a relevant model of XLH for studying early enthesophytes and joint structural damage. Here, we studied the effect of conventional treatment on the development of bone and joint alterations in this mouse model during growth and young adulthood. Mice were supplemented with oral phosphorus and calcitriol injections, following two timelines: (i) from weaning to 3 months of age and (ii) from 2 to 3 months to evaluate the effects of treatment on the development of early enthesophytes and joint alterations, and on changes in bone and joint deformities already present, respectively. We showed that early conventional treatment improved bone microarchitecture, and partially prevented bone and joint complications, but with no noticeable improvement in enthesophytes. In contrast, later administration had limited efficacy in ameliorating bone and joint alterations. Despite the improvement in bone microarchitecture, the conventional treatment, early or late, had no effect on osteoid accumulation. Our data underline the usefulness of the *Hyp* murine model for preclinical studies on skeletal and extraskeletal lesions. Although the early conventional treatment is important for the improvement of bone microarchitecture, the persistence of osteomalacia implies seeking new therapeutic strategies, in particular anti-FGF23 approach, in order to optimize the treatment of XLH.

## Introduction

X-linked hypophosphatemia (XLH) is the most common form of genetic rickets. This rare disease is caused by inactivating mutations in the phosphate-regulating endopeptidase homolog X-linked (*PHEX*) gene, characterized by chronic hypophosphatemia. Impaired function of PHEX leads to elevated levels of phosphaturic fibroblast growth factor 23 (FGF23) resulting in renal phosphate-wasting hypophosphatemia and low levels of calcitriol [1,25(OH)_2_D_3_] via the inhibition of 1α-hydroxylase and the activation of 24-hydroxylase (Kinoshita and Fukumoto, 2018).

Clinically, children with XLH are characterized by progressive skeletal deformities (leg bowing, waddling gait, poor growth, and disproportional short stature), dental abscesses, and craniosynostosis. Adult patients present various symptoms of osteomalacia such as bone pain, insufficiency fractures, and myopathy. In addition, adults may develop hearing loss, odontomalacia, mineralizing enthesopathy, and osteoarthropathy (Linglart et al., 2014). In adult patients with XLH, the aforementioned manifestations significantly reduce quality of life (Che et al., 2016; Steele et al., 2020). We showed that *Hyp* mice, a murine model of XLH, developed early osteoarticular lesions and the severity of these lesions gradually increased over 12 months, demonstrating the relevance of this murine model for osteoarticular preclinical studies (Faraji-Bellee et al., 2020).

Current medical treatment of XLH consists of oral active vitamin D [calcitriol or 1α-(OH)D_3_] and multiple daily doses of phosphate supplements. To optimize the final outcomes (recovery of rickets, normalization of elevated alkaline phosphatase (ALP) levels, growth improvement, restoration of leg deformities, and dental mineralization), treatment should be started as soon as the diagnosis of XLH is made. Supplementation is commonly prescribed from early childhood to the end of growth but is also essential in certain periods of adult life such as pregnancy or breastfeeding, before planned surgical interventions, and in all symptomatic patients with XLH (recurrent dental abscesses, fractures, etc.) (Linglart et al., 2014). Nonetheless, despite this treatment during growth, musculoskeletal symptoms due to enthesopathies and osteoarthritis remain the major manifestations in the clinical progression of XLH. Further, there is a paucity of data on the effects of conventional treatment (i) if started early, on the abnormal bone phenotype in XLH, beyond the traditional goals, and (ii) if started early, on prevention of and/or recovery from osteoarticular manifestations of XLH such as osteoarthritis and enthesopathies. The main limitations of the studies on the effect of conventional treatment in XLH performed so far are small sample sizes and their retrospective cross-sectional observational design that does not take into account the age when the treatment is started.

Therefore, we designed a prospective study in a murine model of XLH (*Hyp* mice) aiming to evaluate whether, if started early in life, conventional treatment is capable of preventing and/or ameliorating the skeletal and extraskeletal manifestations of hypophosphatemia.

## Materials and Methods

### Mice

The *Hyp* mouse model B6.Cg-Phex *Hyp*/J was used in this study. Heterozygous breeding was carried out and tail snips were collected for genotyping. DNA was extracted from the snips using DNeasy Blood and Tissue Kit (Qiagen, France) and the genotype was determined by PCR using primers for the *Phex* gene. Wildtype (WT) and *Hyp* littermate male mice were used in experimental procedures. All experiments were performed with a protocol approved by the Animal Care Committee of the Université de Paris (project agreement 20-008, APAFiS #27827 N°202001171429974). Animals were maintained in accordance with the ethical protocol approved by the Animal Care Committee of French Veterinary Services (DPP Haut de Seine, France: agreement number D9204901). All mice were housed under standard conditions of temperature (23 ± 2°C) with a 12:12 h light-dark cycle and unlimited access to water and standard pelleted food (1.20% calcium and 0.83% phosphorus, rodent diet 3800PMS10, Provimi Kliba, Kaiseraugst, Switzerland).

Two groups of treated *Hyp* mice were carried out in this study (*n* = 6 mice *per* group): (1) to study the effect of long-term treatment on skeletal/extraskeletal manifestations if started early during growth, *Hyp* mice received the conventional treatment during the whole study period from the juvenile stage starting at weaning, which occurs at 3 weeks (W), to the beginning of the mature adult stage which occurs at 3 months (M) of life and (2) *Hyp* mice which received the conventional treatment from M2 (corresponding to the end of juvenile stage) to M3 to study the effect of the conventional treatment on skeletal/extraskeletal manifestations if started later during growth. Both treated groups of *Hyp* mice were compared to control WT and *Hyp* mice, which were not given conventional treatment (*n* = 6 *per* group).

The conventional treatment consisted of intraperitoneal injections of calcitriol 175 pg/g every other day [1,25(OH)2D3, Cayman Laboratory] and phosphate supplementation (phosphate-enriched water 1.93 g phosphate element per liter of beverage, Phosphoneuros). Doses of calcitriol were adjusted once a week according to the animals’ weight.

### *In vivo* Study

#### The Growth Parameters

Growth parameters (body weight and total length) were measured once a week. Precision scales and a graduated ruler were used for weight and length measurements, respectively. Additionally, the length of the rachis was measured on X-ray micro-computed tomography (Micro-CT) images.

#### X-Ray Micro-computed Tomography Analysis

Wildtype and *Hyp* mice were scanned at W3, M2, and M3 using a high-resolution X-ray micro-CT system (Quantum FX Caliper, Life Sciences, Perkin Elmer, Waltham, MA, United States) hosted by the PIV Platform (UR2496, Montrouge, France). Standard acquisition settings were applied (setting the voltage at 90 kV and intensity at 160 mA), and scans were performed with a field of view alternatively focused on the right paw (scan time of 180 s and voxel size of 20 μm^3^), focused on the hip (120 s and 50 μm^3^), or covering the full body (36 s and 236 μm^3^). Micro-CT datasets were analyzed using the built-in multiplanar reconstruction tool, Osirix 5.8 (Pixmeo, Switzerland), to obtain time series of images aligned anatomically for each region of each animal.

Axial and coronal images of the sacroiliac and hip joints, sagittal images of the spine, and axial and sagittal images of the hind paw were reconstructed. The following were evaluated: hip osteoarthritis (defined as the presence of osteophytes on joint margins, narrowing of the joint space or altered shape of the bone ends); enthesopathies (defined as new bone formation at enthesis sites) on the iliac bone, spine or paw; erosion of the sacroiliac joint and periarticular calcification. The analysis was focused on these areas because they are the most frequent sites of structural involvement in adults with XLH. Erosion of the sacroiliac joints was assessed following Faraji-Bellee et al. (2020) protocol, which was developed by rheumatologists specialized in the field of rare bone diseases and bone inflammatory diseases. The reader was blind to the status of the mouse (*Hyp* vs WT) but was aware of the different analysis time points (W3, M2, M3). A semi-quantitative score was established, ranging from 0 (normal) to 3 (most severe feature assessed) for sacroiliac erosions (see Supplementary Table 1).

The angle of dorsolumbar kyphosis of mice was defined for each mouse at W3, M2, and M3. Using sagittal images of mice spines from full-body CT scans, endplate orientations of thoracic and lumbar vertebrae have been marked using ImageJ (Rasband, W.S., ImageJ, U.S. National Institutes of Health, Bethesda, MD, United States^1^ 1997–2016). The apical thoracic vertebrae of the rachis were identified and the angle of kyphosis defined by the means of (1) the tangent to the lower vertebral endplate of the fourth lower vertebra and (2) the tangent of the upper vertebral endplate of the fourth upper vertebra, from the apical vertebra. A script in MATLAB (MATLAB R2012b, The MathWorks Inc., Natick, MA, United States, 2000) was used to measure these angles at each age in each of the mice.

The trabecular bone was analyzed at the distal metaphysis of the femur. The following parameters were used: bone volume/total volume (BV/TV) ratio, trabecular number (TbN), trabecular separation (TbSp), trabecular thickness (TbTh), and trabecular pattern factor (TbPf) (Bouxsein et al., 2010).

### *Ex vivo* Study

#### Murine Bone Tissue Preparation

Bones were fixed overnight at 4°C in 70% ethanol solution and dehydrated in a graded ethanol series. Undecalcified samples were embedded in methyl methacrylate (Merck, Rahway, NJ, United States). Serial sections, 4 μm thick, were cut on a microtome (Polycut E microtome, Leica, Wetzlar, Germany). Series of consecutive sections representative of micro-CT images were stained with von Kossa (5% silver nitrate solution, Sigma-Aldrich, St Louis, MO, United States) and counterstained with toluidine blue (pH 3.8), stained with Masson’s Trichrome (Sigma–Aldrich), Safranin O Lillie’s Trichrome (Sigma–Aldrich).

#### Enzyme Histochemistry

Tartrate-resistant acid phosphatase (TRAP) was used to evaluate osteoclasts by using 2.5 mM naphthol-ASTR-phosphate (Sigma–Aldrich), 0.36 M N–N-dimethyl-formamide (Sigma–Aldrich), and 4 mM salt in pH 5.2 acetate buffer. Non-osteoclastic acid phosphatase activity was inhibited with 100 mM L(+)-tartaric acid (Sigma–Aldrich) added to the substrate solution.

Alkaline phosphatase was used to reveal the layer of osteogenic cells by incubating the sections with naphthol ASTR phosphate (Sigma–Aldrich) and diazonium fast blue RR salt (Sigma–Aldrich) for 30 min at 37°C (pH 9) in the presence of MgCl_2_.

#### Immunohistochemistry

Sections embedded in methyl methacrylate were deplasticized in methyl glycol acetate. After rehydration in a graded ethanol series to pure distilled water, non-specific peroxidases were blocked for 15 min with ortho-periodic acid and background activity was blocked at room temperature using 5% bovine serum albumin (BSA). Sections were then incubated in a humid atmosphere for 12 h at room temperature in a dark chamber with primary antibody against sclerostin (SOST) (R&D Systems, Minneapolis, MN, United States) diluted at 5 μg/mL. Sections were washed and then incubated with polyclonal antigoat immunoglobulin (Dakocytomation) diluted at 1/200 for 1 h at room temperature in a dark chamber. Peroxidase activity was detected using diaminobenzidine (DAB) substrate (Sigma–Aldrich). Control incubations to assess non-specific staining consisted of the same procedure except that the primary antibody was replaced by non-immune serum.

### Statistical Analysis

Statistical analysis was carried out and graphs plotted with GraphPad Prism for Windows, version 7.0. The distribution of variables was tested with Kolmogorov–Smirnov test. The results are expressed as the mean ± SD for continuous variables and comparisons being performed using ANOVA. Data are expressed as median with interquartile range in Figures 4B,C and mean ± SD in Figure 4D using Mann–Whitney and Student’s *t*-test for statistical analysis, respectively. *P*-values of less than 0.05 were considered significant.

## Results

### Effect of Conventional Treatment on Growth and Dorsolumbar Kyphosis

**FIGURE 1:**
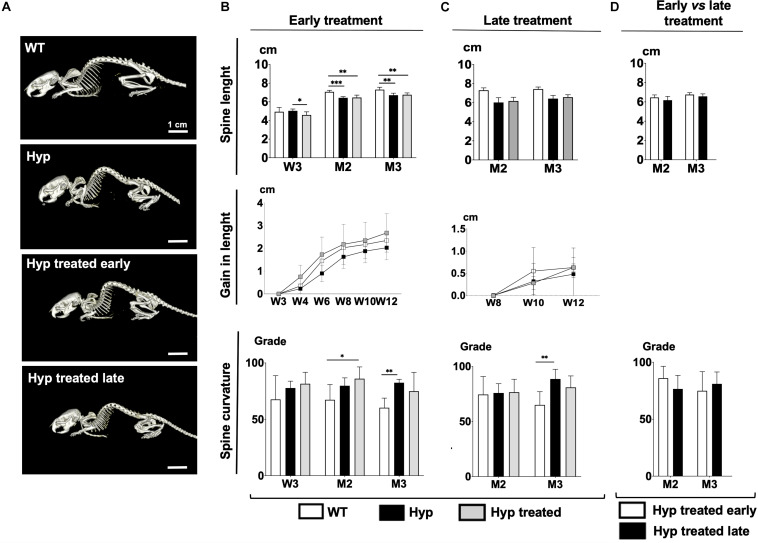
**(A)** Total body images of WT mice, untreated *Hyp* mice, and *Hyp* mice on conventional treatment started early at M3. **(B)** Spine length, gain in length, and spine curvature at baseline (W3), M2 and M3 evaluated with micro-CT in WT mice, untreated *Hyp* mice, and *Hyp* mice on conventional treatment started early. **(C)** Spine length, gain in length, and spine curvature at baseline (M2) and M3 evaluated with micro-CT in WT mice, untreated *Hyp* mice, and *Hyp* mice on conventional treatment started late. **(D)** Spine length and curvature of *Hyp* mice on conventional treatment started early compared to *Hyp* mice on conventional treatment started late. W3, 4, 6, 8, 10, 12: 3, 4, 6, 8, 10, 12 weeks; M2: 2 months; M3: 3 months; *p < 0.05; **p < 0.01; ***p < 0.001.

Seeking to understand the effect of the conventional treatment on parameters such as growth and dorsolumbar kyphosis, we analyzed changes in spine length and changes in dorsolumbar kyphosis (expressed in degrees of curvature) by group (Figure 1).

#### Early Start of Conventional Treatment

At baseline, *Hyp* mice had significantly shorter spine lengths than WT animals (Figure 1B). After starting the treatment, *Hyp* mice in the early treatment group showed much larger gains in length than WT or untreated *Hyp* mice (Figure 1B). On the other hand, micro-CT analysis showed that at M3, WT mice were significantly longer than treated *Hyp* mice, and there were no significant length differences between treated and untreated *Hyp* mice (Figure 1B).

Regarding dorsolumbar kyphosis, spinal kyphosis increased between baseline and the end of the study in untreated *Hyp* mice whereas it decreased over time in the WT group. No significant difference in spine curvature was observed at M3, i.e., at the end of the study, between the *Hyp* mice treated early and WT groups (Figure 1B).

#### Late Start of Conventional Treatment

*Hyp* mice did not show a significant increase in length after initiating the treatment at M2, and no differences in spine length were seen between treated and untreated *Hyp* mice at M3 (Figure 1C). Stature growth was not statistically enhanced by conventional treatment when initiated as an adult. Nevertheless, at M3, *Hyp* mice with treatment showed a gain in length comparable to that in WT animals (Figure 1C).

Regarding dorsolumbar kyphosis, though not statistically significant, spinal kyphosis tended to decrease in *Hyp* mice after initiating treatment, following the same pattern as in the WT group. In contrast, in untreated *Hyp* mice, kyphosis increased over time (Figure 1C).

*Hyp* mice with late treatment showed a spine length and curvature comparable to *Hyp* mice with early treatment (Figure 1D).

### Effect of Conventional Treatment on Bone Microarchitecture

#### Early Start of Conventional Treatment

**FIGURE 2:**
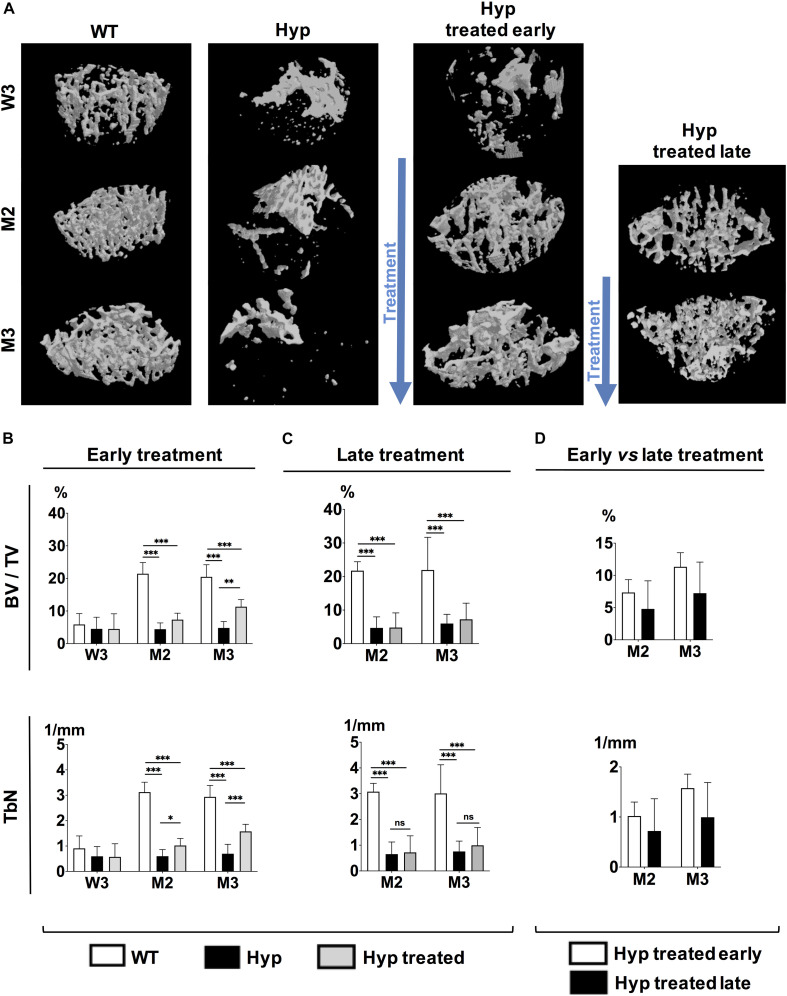
Parameters of bone microarchitecture evaluated with micro-CT in WT mice, untreated *Hyp* mice, and *Hyp* mice on conventional treatment started early and late. **(A)** 3D-images of bone microarchitecture at baseline (W3), M2 and M3 in WT mice, untreated *Hyp* mice, and *Hyp* mice on conventional treatment started early, and at M2 and M3 in WT mice, untreated *Hyp* mice, and *Hyp* mice on conventional treatment started late. **(B)** BV/TV and TbN at baseline (W3), M2 and M3 in WT mice, untreated *Hyp* mice, and *Hyp* mice on conventional treatment started early. **(C)** BV/TV and TbN at baseline (M2) and M3 in WT mice, untreated *Hyp* mice, and *Hyp* mice on conventional treatment started late. **(D)** BV/TV and TbN at M2 and M3 in *Hyp* mice on conventional treatment started early compared to *Hyp* mice on conventional treatment started late. BV/TV, bone volume/total volume ratio; TbN, trabecular number. W3: 3 weeks; M2: 2 months; M3: 3 months; *p < 0.05; **p < 0.01; ***p < 0.001.

3D reconstructed images demonstrated that treated *Hyp* mice had bone microstructure very similar to that in the WT group by the end of study (M3) (Figure 2A). Regarding the parameters of bone quantity, both BV/TV and TbN were significantly higher (*p* < 0.05) by M2 in *Hyp* mice with early treatment than in untreated animals, and this difference was maintained at M3 (Figure 2B). There were no differences in TbTh between WT and *Hyp* mice (treated or not) at any point in the follow-up (Supplementary Figure 1A). Regarding the trabecular connectivity (expressed as TbPf), there were significantly more connections between bone trabecula (*p* < 0.05) in treated than untreated *Hyp* mice, though this difference was only observed at M3 (Supplementary Figure 1A).

#### Late Start of Conventional Treatment

3D reconstructed images demonstrated a slightly greater bone mass in treated than untreated *Hyp* mice, although the bone microstructure in treated *Hyp* mice was far from that in the WT group (Figure 2A). The study of bone microarchitecture showed that there were no statistically significant differences in BV/TV, TbN, TbTh, or TbPf between treated and untreated *Hyp* mice by the end of study (M3) (Figure 2C and Supplementary Figure 1B).

Compared to *Hyp* mice in the early treatment group, *Hyp* mice treated late showed lower BV/TV and TbN at M3, although the statistical significance was not reached (Figure 2D). There were no differences in TbTh and TbPf between *Hyp* mice with early or late treatment groups (Supplementary Figure 1C).

### Effect of Conventional Treatment on Bone and Joint Structural Damages

#### Bone and Joint Alterations in the Axial Skeleton (Sacroiliac Joint)

##### Early start of conventional treatment

**FIGURE 3:**
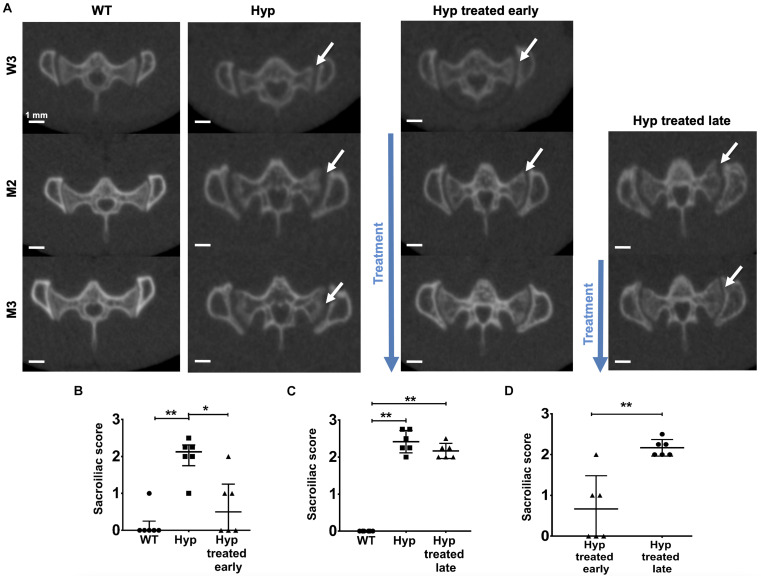
Micro-CT evaluation of sacroiliac joints at W3, M2, and M3 in WT mice, untreated *Hyp* mice and *Hyp* mice on conventional treatment started early or late. **(A)** Multiple erosions and irregular cortical margins of sacroiliac joints are indicated with arrows in untreated *Hyp* mice. In *Hyp* mice on conventional treatment started early, there is improvement in extraskeletal manifestations at M2 and M3, but without complete restoration *ad integrum*. No improvement in extraskeletal manifestations is noticeable in *Hyp* mice on conventional treatment started late. Sacroiliac score evaluated at Micro-CT scans at M3 **(B)** in WT mice, untreated *Hyp* mice, and *Hyp* mice on conventional treatment started early, **(C)** in WT mice, untreated *Hyp* mice, and *Hyp* mice on conventional treatment started late, and **(D)** in *Hyp* mice on conventional treatment started early compared to *Hyp* mice on conventional treatment started late. W3: 3 weeks; M2: 2 months; M3: 3 months; *p < 0.05; **p < 0.01.

Micro-CT images, performed at baseline (W3), showed alterations in the sacroiliac joint in *Hyp* mice, in comparison to WT (Figure 3A). Two out of six untreated *Hyp* mice and two out of six *Hyp* mice in the early treatment group already displayed a high sacroiliac joint score for erosion at baseline (score between 2 and 2.5 out of 3 which means <25% and ≥25% to <50% of the articular surface area affected, respectively) (Supplementary Table 2). At M2 and M3, multiple erosions and an irregular and blurred appearance of the cortical margins were noted in untreated *Hyp* mice, whereas *Hyp* mice started on the conventional treatment early showed fewer erosions and a more regular appearance of the sacroiliac joint, compared to that observed in WT animals (Figure 3A). These results were confirmed by the sacroiliac joint scores for erosion at M3 which were lower in *Hyp* mice with early treatment compared to untreated *Hyp* mice. At M3, three of six *Hyp* mice with early treatment had a mean score of 0, whereas none of untreated Hyp mice had such a mean score (Figures 3B–D and Supplementary Table 2).

##### Late start of conventional treatment

Multiple erosions and irregular and blurred cortical margins of sacroiliac joints were noticed on micro-CT either at M2 or M3 in untreated *Hyp* mice, in comparison to WT animals. *Hyp* mice given treatment, even when initiated late, showed a slight trend of amelioration of these alterations present at M2 before treatment, nonetheless, there were no differences in sacroiliac joint score for erosion between untreated *Hyp* mice and *Hyp* mice with late treatment (Figures 3A,C and Supplementary Table 3).

**FIGURE 4:**
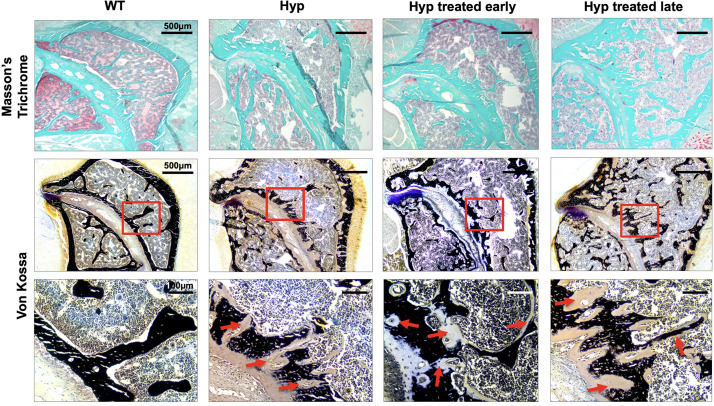
Masson’s Trichrome and Von Kossa staining of sacroiliac joint of 3-month-old WT mice, untreated *Hyp* mice, and *Hyp* mice on conventional treatment started early or late. Masson’s Trichrome and Von Kossa staining showed a weaker mineralization (in dark) of the collagen matrix revealing an enlarged osteoid (red arrows) characteristic of features of osteomalacia bone in *Hyp* mice compared to WT. Despite the conventional treatment, *Hyp* mice treated early or late show an accumulation of osteoid (red arrows).

Compared to *Hyp* mice in the early treatment group, *Hyp* mice treated late had a significant higher score at M3 (Figure 4D and Supplementary Tables 2, 3).

##### Effect of conventional treatment on bone markers

**FIGURE 5:**
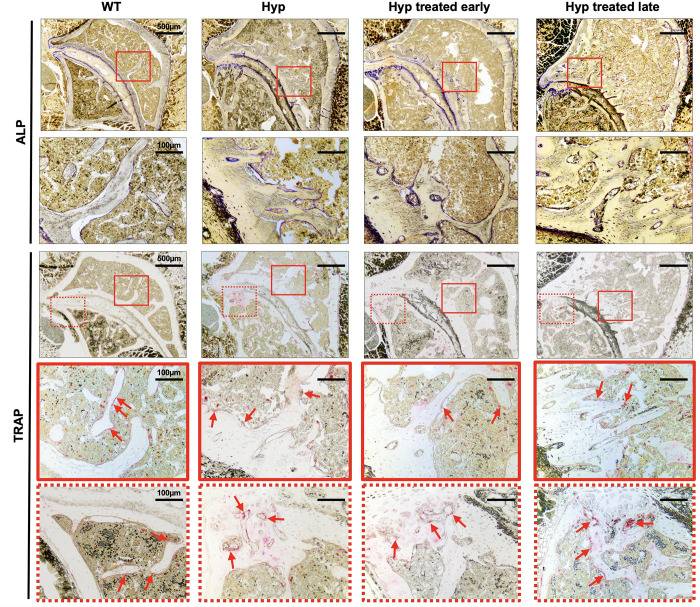
ALP and TRAP enzyme histochemistry of sacroiliac joint of 3-month-old WT mice, untreated *Hyp* mice, and *Hyp* mice on conventional treatment started early or late. The TRAP reaction stained for osteoclast cell activity and the osteogenic layer stained with ALP reaction indicate that both early and late start of conventional treatment in *Hyp* mice hardly changed ALP/TRAP activity. ALP, alkaline phosphatase; TRAP, tartrate-resistant alkaline phosphatase.

To confirm the micro-CT results and study the pathophysiological mechanism, we performed histological analyses of sacroiliac joints of the 3-month-old mice (Figures 4, 5).

Von Kossa and Masson’s Trichrome staining of the sacroiliac joint confirmed altered mineralization with accumulation of osteoid in untreated *Hyp* mice, compared to WT mice. The accumulation of osteoid was still evident in *Hyp* mice with early or late treatment (Figure 4). As expected, *Hyp* mice showed strong ALP staining (a marker of ALP activity), especially at the periphery of the osteoid. In contrast, in untreated *Hyp* mice, TRAP staining indicated that osteoclasts were present but concentrated in large “clusters” in the peripheral zone of sacroiliac joint. Both early and late start of conventional treatment in *Hyp* mice hardly modified ALP/TRAP activity (Figure 5).

We further studied the expression of sclerostin as a marker of differentiated osteocytes and bone turnover. Immunohistochemistry showed sclerostin expression in osteocytes of subchondral bone in WT mice (Supplementary Figure 2). Interestingly, untreated *Hyp* mice and *Hyp* mice treated late showed only faint sclerostin expression. In contrast, in the *Hyp* mice given early treatment, sclerostin expression was somewhat higher, and this finding is suggestive of improved regulation of bone turnover by terminally differentiated osteocytes.

#### Peripheral Enthesophytes (Calcaneus)

##### Early start of conventional treatment

**FIGURE 6:**
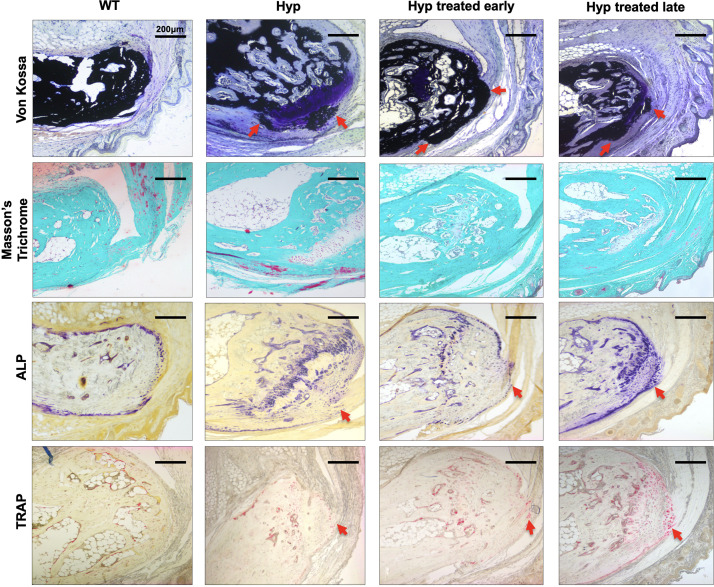
Histological analysis of the calcaneus of 3-month-old WT mice, untreated *Hyp* mice, and *Hyp* mice on conventional treatment started early or late. Masson’s Trichrome and Von Kossa staining of undecalcified sections of calcaneal area confirmed the cellular expansion of mineralizing fibrochondrocytes into Achilles tendon and plantar fascia ligament insertions (red arrows) of the calcaneal tuberosity in *Hyp* mice. ALP and TRAP staining in the area of the enthesophytes at the insertion of the Achilles tendon and plantar fascia ligament was similar in *Hyp* mice that received treatment, either early or late, and in both cases (red arrows) more than that observed in WT mice. ALP, alkaline phosphatase; TRAP, tartrate-resistant alkaline phosphatase.

Micro-CT images of hind paws showed similar features in 3-month-old *Hyp* mice of both treated and untreated groups (Supplementary Figure 3). Histological sections revealed mineralizing fibrochondrocytes expanding into both Achilles tendon and plantar fascia ligament insertions of calcaneal tuberosity in *Hyp* mice (Figure 6).

##### Late start of conventional treatment

The multiple calcaneal enthesophytes present in untreated *Hyp* mice were also seen in *Hyp* mice on conventional treatment started late (Figure 6).

##### Effect of conventional treatment on bone markers

Alkaline phosphatase and TRAP staining in the area of the enthesophytes at the insertion of the Achilles tendon and plantar fascia ligament was similar in *Hyp* mice that received treatment, either early or late, and in both cases more than that observed in WT mice (Figure 6). That is, these alterations persisted in *Hyp* mice regardless of early conventional treatment. Neither early nor late conventional treatment seems able to prevent excessive bone mineralization at the Achilles tendon (enthesopathies) and restore ALP and TRAP activity (Figure 6).

In WT mice, sclerostin labeling was not observed in the Achilles tendon. In contrast, labeling was observed in both untreated and treated (early or late) *Hyp* mice at the insertion of the tendon, corresponding to the area where enthesopathies develop (Supplementary Figure 2).

## Discussion

The main focus of conventional treatment (phosphate supplements and active vitamin D) is growth restoration, and the impact of this type of treatment on XLH manifestations has been poorly investigated after the end of growth. In this context, we studied its effect on the main manifestations of XLH, in particular, skeletal features. Our study is the first to demonstrate that conventional treatment of XLH started early significantly improves bone microarchitecture and sacroiliac joint lesions but has little effect on enthesopathies assessed at the calcaneus. Empirically, it is assumed that current medical treatment of XLH should be started as early as possible to optimize final clinical outcomes in children (Linglart et al., 2014; Haffner et al., 2019). Nonetheless, this has yet to be proven in prospective studies. We have demonstrated that early conventional treatment has a significantly positive effect on impaired skeletal development. Micro-CT analysis showed that *Hyp* mice with early treatment showed a progressive increase in parameters of bone quantity (BV/TV, TbN) and structure (TbPf) resulting in a bone microarchitecture similar to WT mice. Interestingly, patients with XLH show compromised trabecular microarchitecture despite conventional treatment received since childhood (Cheung et al., 2013; Shanbhogue et al., 2015; Colares Neto et al., 2017). The major limitation of these studies is their retrospective design without taking into account age at the start or the duration of conventional treatment.

Importantly, our histological analyses showed a persistence of osteoid accumulation in treated *Hyp* mice by Von Kossa staining. Regardless of improvement of bone microarchitecture, neither early nor late treatment cured osteomalacia. However, restoration of sclerostin expression at the sacroiliac joint in *Hyp* mice in the early treatment group suggested that terminally differentiated osteocytes may regulate bone turn over, even if ALP and TRAP activities presented similar features in treated and untreated *Hyp* groups.

The effect of conventional treatment on skeletal manifestations depends on the bone involved. A significant reduction in the sacroiliac alterations was demonstrated in *Hyp* mice treated early, compared to *Hyp* mice treated late. Regarding the peripheral skeleton, conventional treatment, both early and late, has little effect on enthesopathies. *Hyp* mice in both treatment groups had persistent heel enthesophytes at the end of the study. The ALP expression in Achilles tendon in treated *Hyp* mice ultimately confirmed the expansion of mineralizing fibrochondrocytes in ligaments. Interestingly, we noticed sclerostin expression by fibrochondrocytes at the tendon of *Hyp* mice treated early. These results are concordant with Karaplis et al. (2012) findings and also the clinical study by Connor et al. (2015), which also demonstrated the limited effect of conventional treatment on enthesopathies in patients with XLH. From a pathophysiological point of view, hyperplastic fibrocartilaginous chondrocytes in the tendon are independent of improved mineralization of the bony part at the calcaneus. In this condition, newly mineralized bone is likely not strong enough to prevent the expansion of mineralizing fibrocartilaginous chondrocytes within the enthesis at the points of mechanical strain, a process that may be a compensatory mechanism to respond to biomechanical properties of poorly mineralized bone.

Nowadays, the mechanism of early osteoarthritis and enthesopathies in XLH is poorly understood. The degenerative osteoarthropathy principally depends on the accumulation of unmineralized immature bone (Liang et al., 2011; Wei and Bai, 2016). Additionally, high levels of FGF23 may promote the Wnt/ß-catenin pathway in chondrocytes which activates the genes responsible for increased chondrocyte differentiation and osteoarthritis progression. Consequently, low expression of inhibitors of the Wnt/ß-catenin pathway such as sclerostin is usually associated with the promotion of cartilage degradation (Meo Burt et al., 2018). Indeed, the results of our study confirm that accumulation of osteoid in the subchondral bone and lack of sclerostin expression are associated with the appearance and progression of numerous erosions at the sacroiliac joint in non-treated *Hyp* mice. On the contrary, sclerostin expression was restored in *Hyp* mice treated early, suggesting a link with the reduction of sacroiliac bone erosion in this group compared to Hyp mice with late treatment.

Early conventional treatment did not completely restore spine length in *Hyp* mice. Conventional treatment significantly increased the gain in length in *Hyp* mice treated early or late, though it did not have a significant impact on final length. In fact, we found no significant differences in length as assessed by micro-CT between non-treated and treated *Hyp* mice (early or late) at the end of the experiment. These results are in line with other studies performed in children with XLH. Specifically, healing active rickets promotes growth and after 2 years of successful treatment growth velocity is restored to its maximal potential in the majority of patients; however, 25–40% of patients with well-controlled XLH show linear growth failure despite optimized treatment (Linglart et al., 2014). It may be explained by several reasons. First, linear growth depends on bone mineralization and also on the cartilage growth plate (Fuente et al., 2018). Partial growth restoration might result from a poor effect of the conventional treatment on growth plate maturation. Second, the conventional treatment, being a supplementary treatment, does not have influence on other players involved in XLH pathogenesis such as high levels of FGF23 (Carpenter et al., 2010; Zhukouskaya et al., 2020) or accumulation in the extracellular matrix of other proteins or peptides (osteopontin, ASARM peptides, etc.) (Salmon et al., 2013, 2014; Coyac et al., 2018).

The major strength of our study lies in its clinical implications. This is the first preclinical research demonstrating the beneficial effects of early conventional treatment in a well-designed prospective study, the findings underlining the importance of this treatment in the management of XLH. Our results were confirmed by several different methods, from clinical manifestations to radiological and histological analysis. Overall, the data provided highlight the usefulness of the *Hyp* murine model for preclinical studies on skeletal and osteoarticular lesions.

## Conclusion

We have shown that conventional treatment given since an early stage improves bone microarchitecture and prevents joint erosions, though it does not have a notable effect on the formation of enthesophytes. Despite the improvement of bone microarchitecture, the persistence of osteomalacia implies seeking new therapeutic strategies. Further studies are needed to compare these outcomes with the potential benefits of new therapies such as anti-FGF23 to improve the treatment of XLH.

## Data Availability Statement

The raw data supporting the conclusions of this article will be made available by the authors, without undue reservation.

## Ethics Statement

All experiments were performed with a protocol approved by the Animal Care Committee of the Université de Paris (project agreement 20-008, APAFiS #27827 N°202001171429974). Animals were maintained in accordance with the ethical protocol approved by the Animal Care Committee of French Veterinary Services (DPP Haut de Seine, France: agreement number D9204901).

## Author Contributions

AL, CC, and CB contributed to the design of the experiments. AC, VVZ, SH, JS, LS, CM-R, KB, AL, CC, and CB performed and analyzed the experiments. VVZ and CB wrote the manuscript with contributions from all authors. All authors reviewed and approved the final version of the manuscript.

## Conflict of Interest

The authors declare that the research was conducted in the absence of any commercial or financial relationships that could be construed as a potential conflict of interest.

## Footnotes

1 https://imagej.nih.gov/ij/
