# Supplementary material for: Impact of Early Conventional Treatment on Adult Bone and Joints in a Murine Model of X-Linked Hypophosphatemia
Source: Front Cell Dev Biol. 2021 Feb 18;8:591417. doi: 10.3389/fcell.2020.591417 (PMC7930336; doi:10.3389/fcell.2020.591417)
Supplement: Supplementary file 1 [file Presentation_1.pdf]

Supplementary Material

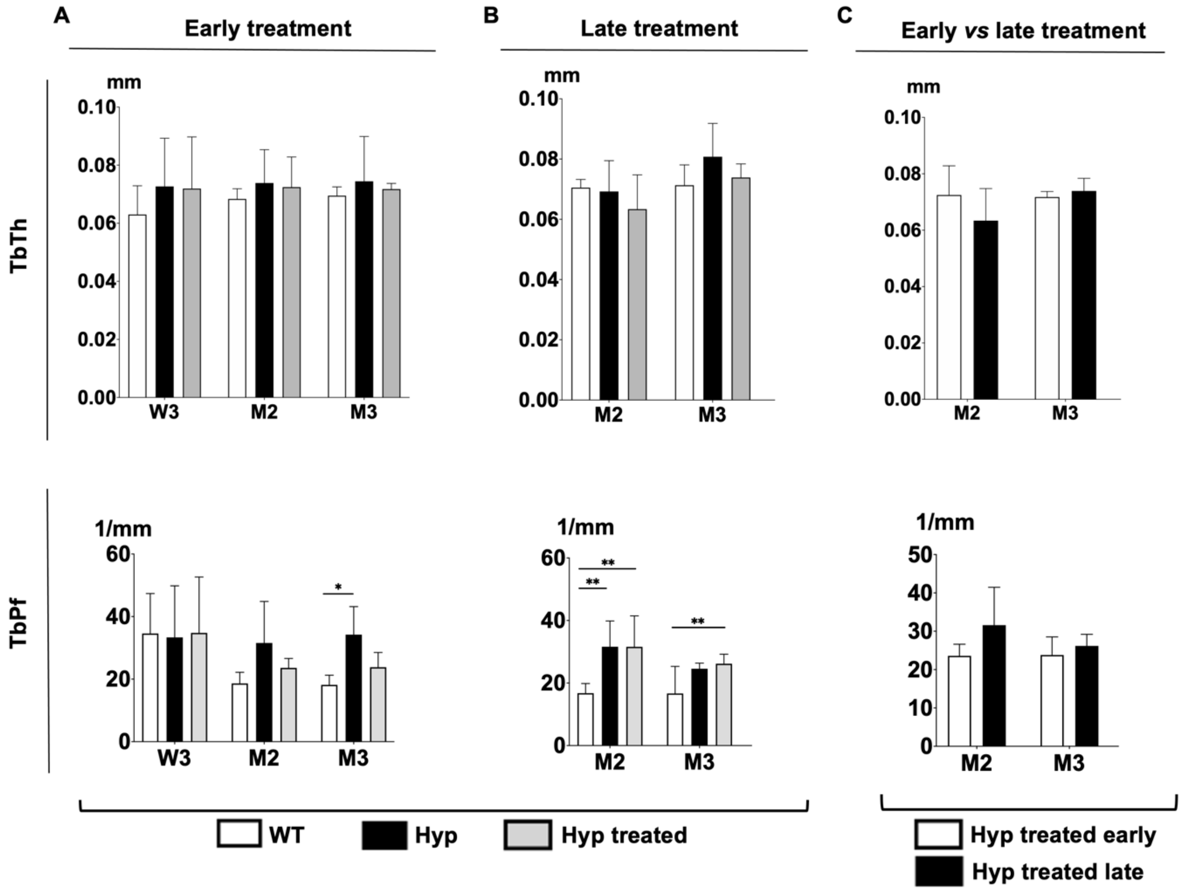

SUPPLEMENTARY FIGURE 1. (A) TbTh and TbPf at baseline (W3), M2 and M3 in WT mice, untreated *Hyp* mice and *Hyp* mice on conventional treatment started early. **(B)** TbTh and TbPf at baseline (M2) and M3 in WT mice, untreated *Hyp* mice and *Hyp* mice on conventional treatment started late. **(C)** TbTh and TbPf at M2 and M3 in *Hyp* mice on conventional treatment started early compared to *Hyp* mice on conventional treatment started late. TbTh: trabecular thickness; TbPf: trabecular bone pattern factor; W3: 3 weeks; M2: 2 months; M3: 3 months; * $p < 0.05$; ** $p < 0.01$.

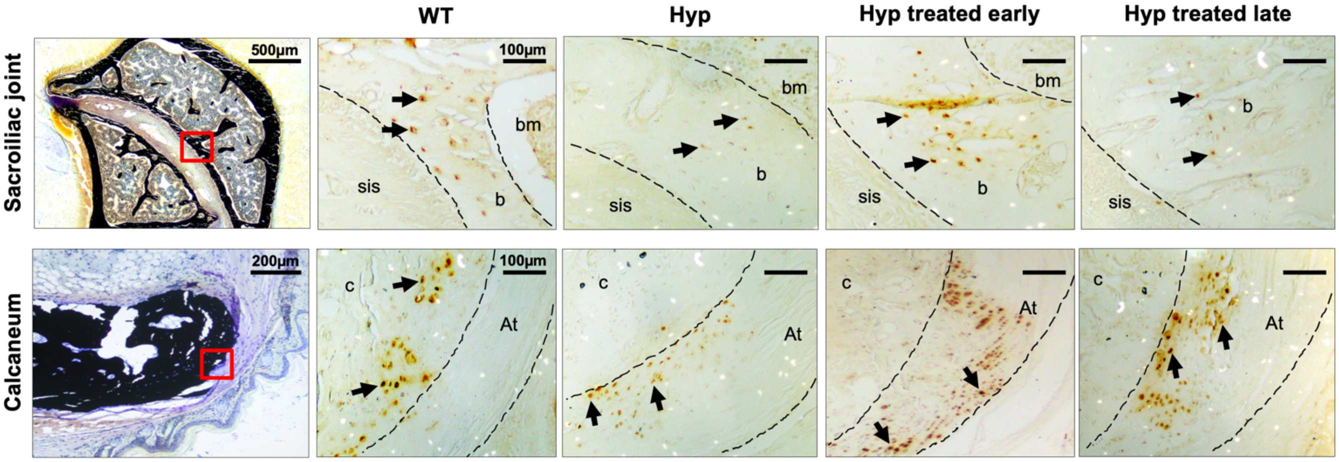

SUPPLEMENTARY FIGURE 2. Immunohistochemistry for sclerostin expression at sacroiliac joint and calcaneus in 3-month-old WT mice, untreated *Hyp* mice, and *Hyp* mice on conventional treatment started early or late: sclerostin staining (arrows) in subchondral bone at sacroiliac joint and in mineralizing fibrochondrocytes of Achilles tendon. b: bone; sis: sacroiliac space; bm: bone marrow; c: calcaneum; At: Achilles tendon.

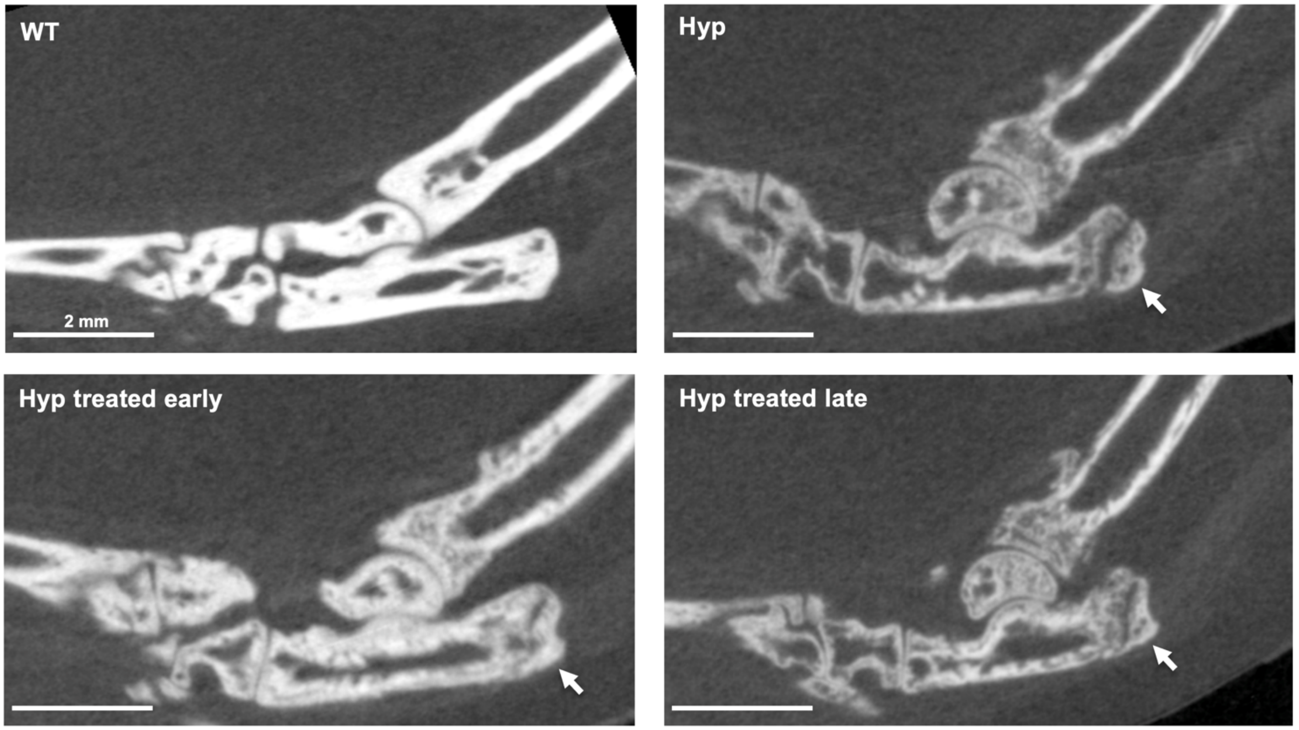

SUPPLEMENTARY FIGURE 3. Micro-CT images of hind paws in 3-month-old WT mice, untreated *Hyp* mice and *Hyp* mice on conventional treatment started early or late.

SUPPLEMENTARY TABLE 1. Scoring grid of erosions.

Variables	Score	Description
Erosion of the sacroiliac joints	**0**	normal
	**1**	doubtful
	**2**	< 25% of the articular surface area affected
	**2.25**	≥ 25% to < 50% of the articular surface area affected
	**2.5**	≥ 50% to < 75% of the articular surface area affected
	**2.75**	≥ 75% to < 100% of the articular surface area affected
	**3**	all the articular surface area affected

SUPPLEMENTARY TABLE 2. Sacroiliac joint score for erosion in *Hyp* mice on conventional treatment started early compared to untreated *Hyp* mice and WT mice.

**Age**	**W3**						**M3**					
**Mice #**	**#1**	**#2**	**#3**	**#4**	**#5**	**#6**	**#1**	**#2**	**#3**	**#4**	**#5**	**#6**
**WT**	0	1	0	1	0	0	0	0	1	0	0	0
**Hyp**	0	1	2.25	1	1	2	2	2.25	2.25	1	2.25	2
**Hyp treated early**	0	0	0	1	2.25	1	1	0	0	2	1	0

W3: 3 weeks of age; M3: 3 months of age.

SUPPLEMENTARY TABLE 3. Sacroiliac joint score for erosion in *Hyp* mice on conventional treatment started late compared to untreated *Hyp* mice and WT mice.

**Age**	**M2**						**M3**					
**Mice #**	**#1**	**#2**	**#3**	**#4**	**#5**	**#6**	**#1**	**#2**	**#3**	**#4**	**#5**	**#6**
**WT**	0	0	0	0	0	0	0	0	0	0	0	0
**Hyp**	2.25	2.75	2.75	2.5	2.75	2.5	2.25	2.5	2.75	2.25	2.75	2
**Hyp treated late**	2.75	2.25	2.5	2.5	2.5	2.25	2.25	2	2.5	2.25	2	2

M2: 2 months of age; M3: 3 months of age.
